# The Use of Wearable Activity Monitors to Measure Upper Limb Physical Activity After Axillary Lymph Node Dissection and Sentinel Lymph Node Biopsy

**DOI:** 10.1245/s10434-023-13966-7

**Published:** 2023-07-28

**Authors:** Nur Amalina Che Bakri, Richard M. Kwasnicki, Emmanuel Giannas, Luqman Tenang, Naairah Khan, Catharina Moenig, Zoha Imam, Kieran Dhillon, Hutan Ashrafian, Ara Darzi, Daniel R. Leff

**Affiliations:** 1 Department of Surgery and Cancer, Imperial College London, London, UK; 2 Imperial College Healthcare NHS Trust, St. Mary’s Hospital, London, UK

## Abstract

**Background:**

We suspect that morbidity from both sentinel lymph node biopsy (SLNB) and axillary lymph node dissection (ALND) has been inadequately evaluated to date. Current methodologies are subjective and susceptible to bias. Objective assessment using wearable activity monitors (WAMs) would allow quantitative analysis of recovery by measuring physical activity (PA) and could provide evidence for axillary de-escalation.

**Patients and Methods:**

A prospective, single center, observational study was conducted from February 2020 to May 2022. Consecutive patients undergoing breast and/or reconstructive surgery and axillary surgeries were identified from the operating schedules. Patients wore WAMs for an average of 3 days prior to surgery and up to 2 weeks following surgery. In total, 56 patients with breast cancer were recruited, of whom 35 underwent SLNB and 21 ALND.

**Results:**

Patients who underwent ALND experienced significantly worse PA compared with those who underwent SLNB in week 2 (median 66.4% versus 72.7%, *p* = 0.015). Subgroup analysis revealed significantly lower PA in simple mastectomy (Mx)-ALND versus Mx-SLNB (median 90.3% versus 70.5%, *p* = 0.015) in week 2. The PA for SLNB did not return to baseline at 2 weeks after surgery.

**Conclusions:**

Compared with SLNB, ALND results in a lower PA level in week 2. The findings also indicate that SLNB has a protracted effect on PA levels, which extend to 2 weeks postoperatively. Monitoring recovery objectively following breast cancer surgery provides patients and surgeons with more information regarding the predicted outcomes of their surgery, which can drive the development of a personalized rehabilitation program.

**Supplementary Information:**

The online version contains supplementary material available at 10.1245/s10434-023-13966-7.

Axillary lymph node dissection (ALND) has historically been performed routinely to treat metastases of the primary lymphatics of the breast, which is widely recognized as a significant prognostic factor for survival and recurrence.^1^ The advent of sentinel lymph node biopsy (SLNB) using a radio-guided technique for staging of the axilla^2^ has led to a reduction in postoperative morbidity due to fewer ALND procedures.^3^

Breast surgery can result in an array of postoperative complications including pain, seroma, lymphedema and reduced range of motion (ROM).^3,5^ These sequelae are associated with a significant reduction in quality of life (QoL), with limitations in upper limb (UL) function, often impacting patients’ years postoperatively.^3^ On the basis of the recent meta-analysis by our group,^3^ the prevalence of lymphedema after ALND is higher than previously estimated, particularly in the long term, where the prevalence was 23.6%.^3^ In comparison with SLNB, ALND has a greater risk of UL morbidities.^3,6–10^

The mistaken belief that impairments resolve without intervention over time may have contributed to a diminished emphasis on monitoring disabilities, particularly by objective means.^11^ We believe that the morbidity associated with SLNB and ALND has not been effectively examined using the current approaches, which are mostly subjective and susceptible to bias.^3,5^ Various methods to capture postoperative morbidity have been previously used, such as QoL questionnaires, self-reported outcomes in conjunction with more functional measures such as the Disabilities of the Arm, Shoulder, and Hand (DASH) questionnaire, arm measurements, and grip strength.^3,5,12^ However, objective measurement of physical activity (PA) and arm movements using current technology after breast surgery is lacking. A lack of standardization in the measurement of UL complications has been highlighted in a recent systematic review,^3^ as has the requirement for quantitative and validated outcome measures.^3^ Our team has recently validated the use of wearable activity monitors (WAMs) as an objective measurement tool of UL activity in breast cancer cohort.^13^

The ALMANAC trial measured shoulder function and lymphedema objectively using a goniometer and tape measurement, respectively.^14^ Nevertheless, measurements were deemed problematic due to a high degree of between-observer variation.^14^ WAMs, meanwhile, could reduce measurement and operator variability and have been validated to objectively monitor postoperative UL activity levels in a non-invasive and unbiased manner.^13^ They may be used to ascertain an approximate return to baseline PA and further allow for better delineation of the recovery process between different operations and interventions. Several commercially available products such as the FitBit are able to determine calorie expenditure by measuring movement at the wrist.^15^ Following this concept, WAMs can be worn on both wrists to assess arm and shoulder function post-surgery and provide discriminative information between limbs.

Objective longitudinal assessment of functional UL recovery after ALND and SLNB allows for determination of standard recovery curves and further optimizes patient outcomes by encouraging PA. Such interventions are relevant as evidence begins to strengthen the association between PA and survivorship after breast surgery.^16^ We aimed to use WAMs to investigate differences in physical recovery between ALND and SLNB. We hypothesized that: (1) ALND would experience a greater reduction in PA compared with SLNB in the first 2 weeks postoperatively, (2) PA level of patients who had SLNB would return to baseline level at 2 weeks after surgery, and (3) there would be a significant reduction of light- and moderate-intensity activities from preoperative to postoperative for both SLNB and ALND.

## Patients and Methods

### Study Participants

This was a prospective observational cohort study focusing on the assessment of PA after breast surgery. The study was approved by the National Research Ethics Committee (ref. 15/LO/1038), and the study methods were submitted to the ClinicalTrial.gov registry (NCT03635723). All participants recruited provided informed written consent. Patients who underwent breast/reconstruction and axillary surgeries at Imperial College Healthcare NHS Trust were identified from the operating schedules and tumor boards from February 2020 to May 2022. SLNB and ALND cohorts, regardless of different breast surgeries, were chosen for general comparison. Simple mastectomy (Mx)-SLNB versus Mx-ALND and deep inferior epigastric perforator (DIEP)-SLNB versus DIEP-ALND were chosen for subgroup analyses. Inclusion and exclusion criteria can be found in Supplementary Table 1.

### Study Protocol

The study protocol and analyses were validated in our previous study.^13^ The full protocol is provided in Supplementary Method 2. Patients were fitted with wrist-worn sensors (AX3; Axivity, Newcastle upon Tyne, UK) which are triaxial accelerometers that are commercially available and permit manual calibration, full data download, and analysis using Open Movement Graphical User Interface OMGUI (version 1.0.0.37). WAMs were worn on both wrists daily for an average of 3 days^17–19^ prior to surgery and for up to 2 weeks after surgery. WAMs were worn 24 h per day, although patients were permitted to remove them for sleeping and showering. Patients were informed that the sensors measured arm activities, but no exercise goals were provided. However, in our unit, it is routine procedure to provide all patients with standard postoperative arm mobility advice and a breast cancer care pamphlet on postoperative exercises. The pamphlet is accessible at www.breastcancernow.org. Patients undergoing ALND are not required to proactively wear a compression sleeve. Patients who develop arm swelling or reduced ROM are referred to see a lymphedema nurse or physiotherapist.

Patients completed Disability of the Arm, Shoulder, and Hand (DASH) and EuroQol-5D-5L (EQ-5D-5L) questionnaires before (at the time of recruitment) and after (week 1 and week 2) surgery. Pain score was inferred from DASH questionnaires.

SVM was calculated by OMGUI software using the equation, SVM − 1 = sqrt(*x*^2^ + *y*^2^ + *z*^2^) − 1.^13^ The preoperative SVM level was determined by calculating the average of SVM for days prior to surgery. PA was determined for each postoperative day (POD) by calculating the percentage of the average preoperative SVM level (SVM for each postoperative day/mean preoperative SVM × 100) and comparing the level of activities between treatment groups at week 1 and week 2.^13^ PA was divided into sedentary (1.5), light (1.5–4), moderate (4–7), and vigorous (> 7) intensity on the basis of metabolic equivalent tasks.^20^

### Outcomes

The primary outcome of the study was both PA level and intensity following either SLNB or ALND (regardless of the type of breast procedure). Planned subgroup analysis of PA level was performed comparing Mx-SLNB versus Mx-ALND and DIEP-SLNB versus DIEP-ALND. The secondary outcome was the DASH score and the pain score correlation with the PA level.

### Statistical Analyses

Nonparametric statistical significance tests were performed as the data were non-normally distributed (Shapiro–Wilk test). Using the Wilcoxon signed-rank test, between-group differences in longitudinal regain-of-function data were analyzed. The Mann–Whitney U test was used to assess differences in arm activity between SLNB and ALND groups. The Friedman test with Bonferroni correction was performed to analyze the intensity reduction from preoperative to postoperative level. A sensitivity analysis was performed matching breast and/or reconstructive surgery across different axillary procedures. A *p*-value of less than 0.05 was set as the threshold for statistical significance. Data were analyzed using version 26 of IBM SPSS Statistics (IBM, Armonk, New York, NY, USA). This is the first study using WAMs to develop hypotheses and determine objective PA of the arm following SLNB and ALND, which can then be used to calculate sample size.

## Results

**Fig. 1:**
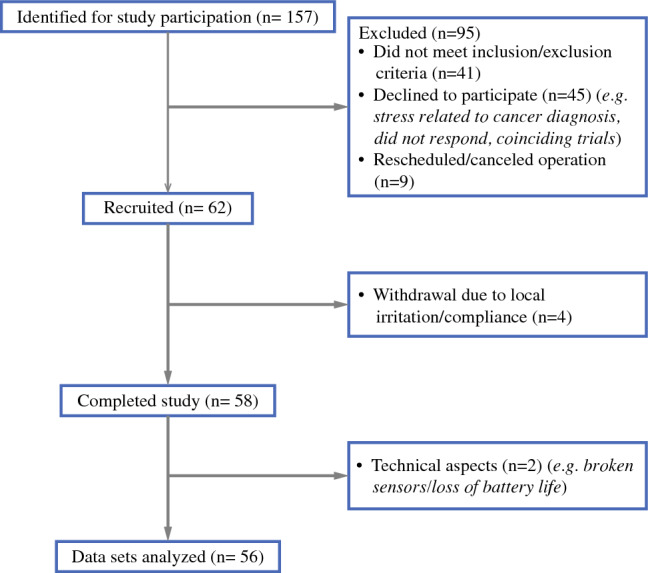
Participant flow diagram

Between February 2020 and May 2022, a total of 157 patients (Fig. 1) at Imperial College Healthcare NHS Trust were identified as potentially eligible from the electronic medical records. The study was halted from March 2020 to July 2020 due to the coronavirus disease 2019 (COVID-19) pandemic. Forty-one patients did not meet the inclusion and exclusion criteria and 45 patients declined to participate for reasons including stress related to cancer diagnosis, coinciding trials, and declined without reason. Nine patients had to be rescheduled or had their surgeries canceled. Two patients initially recruited were subsequently excluded due to technical reason (e.g., broken sensors). Four patients withdrew from the study (non-compliant, local irritation). In total, 56 patients provided data for analysis (35 SLNB, 21 ALND) (Table 1). None had preexisting restricted shoulder ROM, movement disorder, or limb injuries that might impair mobility.

**Table 1:** Demographics of study population (*n* = 56) (means presented with standard deviations and numbers presented with whole number percentage of total population in each group)

Characteristics	SLNB (*n* = 35)	ALND (*n* = 21)		*p*-Value
Mean age, years (SD)	59 (10.8)	56 (11)		0.463
Sex ratio (M:F)	1:34	0:21		0.629
Comorbidities, *n* (%)	23 (65.7)	13 (61.9)		0.372
Mean BMI (SD)	27.1 (6.5)	30.8 (6.3)		0.073
Handedness ratio (R:L)	34:1	21:0		–
Race/ethnicity, *n* (%)				
White British	11 (31.4)	2 (9.5)		0.338
White Irish	2 (5.7)	1 (4.8)		
White and any other white background	2 (5.7)	3 (14.3)		
Asian or Asian British, Indian/Pakistani	1 (2.9)	1 (4.8)		
Asian, any other Asian background	1 (2.9)	1 (4.8)		
Black or Black British African	3 (8.6)	2 (9.5)		
Black or Black British Caribbean	1 (2.9)	1 (4.8)		
Black or any other Black background	0 (0)	1 (4.8)		
Mixed, white and Black Caribbean	0 (0)	1 (4.8)		
Other, any other ethnic group	14 (40)	8 (38.1)		
Stage of cancer, *n* (%)				
Stage 0	5 (14.3)		1 (4.8)	0.003
Stage 1	25 (71.4)		6 (28.6)	
Stage IIA and IIB	4 (11.4)		10 (47.6)	
Stage IIIA–IV	1 (2.9)		4 (19)	
Unknown	0 (0)		0 (0)	
Cancer type, *n* (%)				
Ductal carcinoma in situ	6 (17.1)		0 (0)	0.028
Invasive ductal carcinoma	26 (74.3)		16 (76.2)	
Invasive lobular carcinoma	2 (5.7)		1 (4.8)	
Invasive mucinous carcinoma	0 (0)		1 (4.8)	
Other	1 (2.9)		3 (14.3)	
Previous breast surgery, *n* (%)	6 (17.1)		6 (28.6)	0.317
Patients with drain, *n* (%)	21 (60)		19 (90.5)	0.024
Drain in situ in axilla/breast, mean days (SD)	3.71 (3.9)		9.8 (6.1)	< 0.001
Length of stay, days (SD)	1.2 (1.6)		2.3 (2.1)	0.05
Advice about exercise, *n* (%)	35 (100)		21 (100)	–
Compliance to analgesia, *n* (%)	35 (100)		21 (100)	–
Type of breast surgery/reconstruction, *n* (%)				
Partial mastectomy	11 (31.4)		1 (4.8)	0.211
Mastectomy	10 (28.6)		10 (47.6)	
DIEP	6 (17.1)		3 (14.3)	
TRAM	1 (2.9)		2 (9.5)	
TUG	1 (2.9)		0 (0)	
Implant	6 (17.1)		1 (4.8)	
No breast surgery	0 (0)		4 (19)	
Operation laterality ratio (R:L:BL)	17:16:2		11:9:1	0.772
Neoadjuvant therapy, *n* (%)				
Radiotherapy	1 (2.9)		1 (4.8)	0.014
Chemotherapy	7 (20)		11 (52.4)	
Hormone therapy	2 (5.7)		2 (9.5)	
Monoclonal antibodies	1 (2.9)		1 (4.8)	
Adjuvant therapy while wearing WAMs, *n* (%)				
Radiotherapy	0 (0)		0 (0)	0.145
Chemotherapy	0 (0)		0 (0)	
Hormone therapy	1 (2.9)		3 (14.3)	
Complications, *n* (%)				
Seroma	2 (5.7)		2 (9.5)	0.995
Infected hematoma	1 (2.9)		0 (0)	
Abscess	0 (0)		1 (4.8)	
Cording	1 (2.9)		0 (0)	

DIEP deep inferior epigastric perforator, TUG transverse upper gracilis, TRAM transverse rectus abdominal muscle

### Main Findings

#### Surgically Treated Side versus Control Arm

**Fig. 2:**
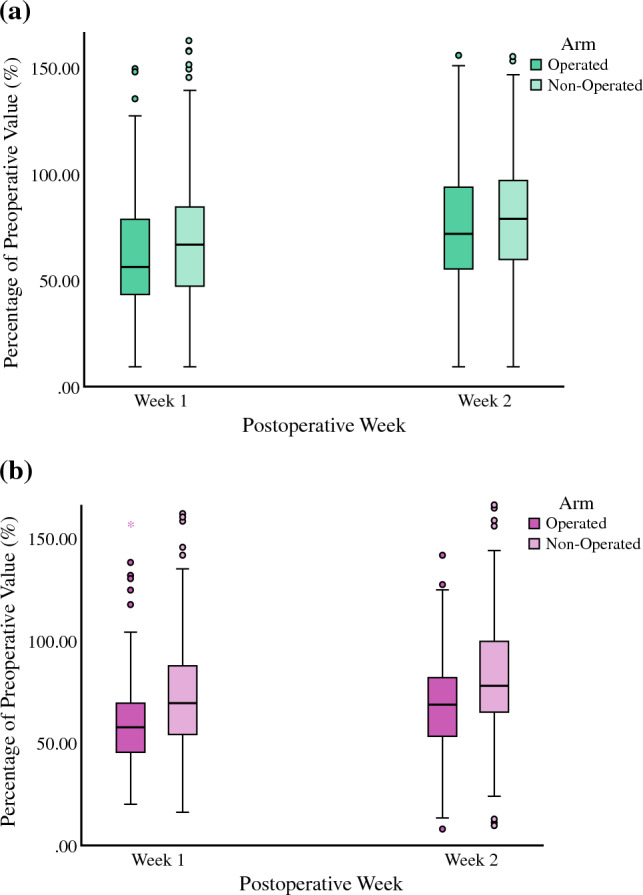
**a** Surgically treated side versus control arm expressed as percentage (%) of preoperative level at week 1 and week 2 after sentinel lymph node biopsy (SLNB). **b** Surgically treated side versus control arm expressed as percentage (%) of preoperative level at week 1 and week 2 after axillary lymph node dissection (ALND)

As illustrated in Fig. 2 (panels a and b), greater PA level (as a percentage of preoperative level) was observed in the control arm compared with the surgically treated side in both SLNB and ALND groups in week 1 (median SLNB 66.9% versus 56.1.1%, *p* = 0.006; median ALND 69.4% versus 57.7%, *p* < 0.001) and week 2 (median SLNB 79% versus 71.8%, *p* = 0.283; median ALND 79.7% versus 68.9%, *p* < 0.001), respectively.

#### Surgically Treated Side Only and Overall Activity Levels

**Fig. 3:**
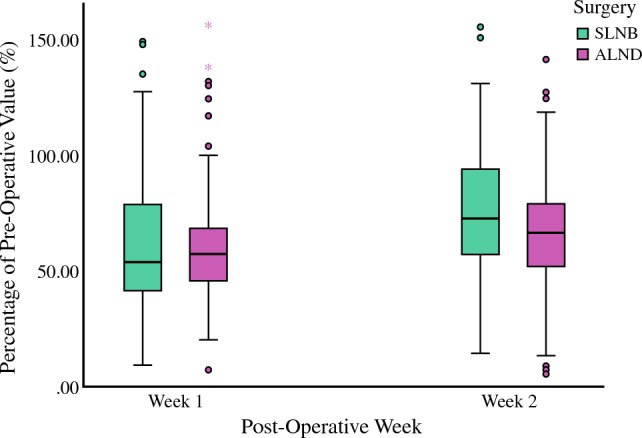
Surgically treated side only expressed as percentage (%) of preoperative level at week 1 and week 2 comparing sentinel lymph node biopsy (SLNB) with axillary lymph node dissection (ALND)

When comparing activities of the surgically treated side only, ALND group experienced significantly lower PA level compared with SLNB group in postoperative week 2 (median 66.4% versus 72.7%, *p* = 0.015) (Fig. 3). There was no significant difference (median 57.7% versus 56.1%, *p* = 0.916) in the PA level in week 1 between ALND and SLNB. When comparing the overall activity levels (combined activity levels of the surgical and the non-surgical side) between ALND and SLNB, there was no significant difference in week 1 and week 2.

### Subgroup Analyses

Subgroup analysis was performed to compare surgically treated side of Mx-SLNB versus Mx-ALND. Compared with Mx-SLNB, PA level was significantly worse in Mx-ALND in week 2 (median 90.3% versus 70.5%, *p* = 0.015) but not in week 1 (median 68.8% versus 57.4%, *p* = 0.274). Seven patients in SLNB group and eight patients in ALND group had drains inserted. Similar findings were observed when patients without drains were excluded in subgroup analysis (Supplementary Table 3).

Interestingly, no significant difference in PA was observed comparing DIEP-SLNB and DIEP-ALND in week 1 (median 46% versus 52.6%, *p* = 0.79) or week 2 (median 50.7% versus 53.5%, *p* = 0.86).

### Intensity Analysis

**Fig. 4:**
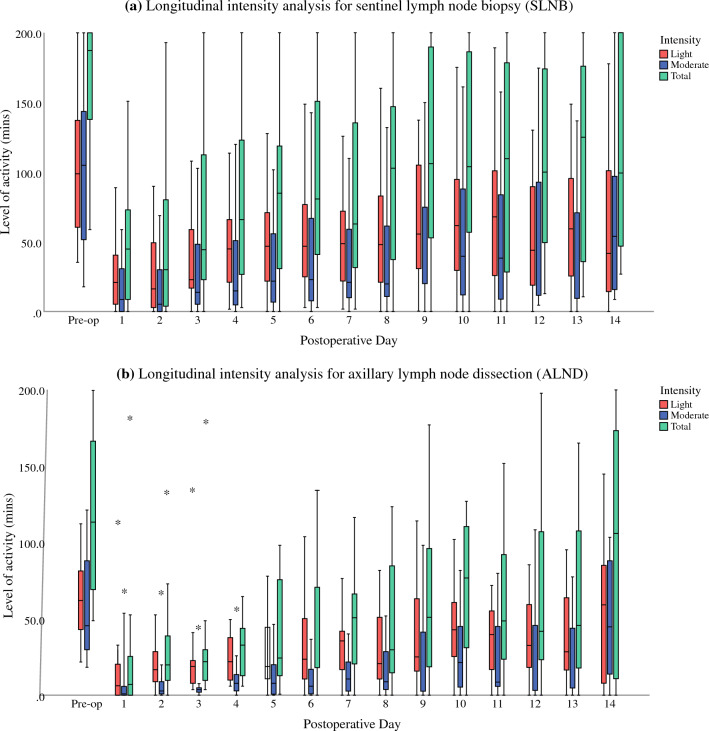
**a** Level of activity spent in light and moderate intensity (min/day) for patients who underwent SLNB at week 1 and week 2; sedentary and vigorous intensity are not shown, total activity (light + moderate + vigorous). **b** Level of activity spent in light and moderate intensity (min/day) for patients who underwent ALND at week 1 and week 2; sedentary and vigorous intensity are not shown, total activity (light + moderate + vigorous)

On average, patients who underwent ALND and SLNB spent 95.8% and 92.8% of their time in sedentary state over 24 h, respectively. A marked decline in activity was observed in both SLNB and ALND groups in the initial postoperative period, with a gradual increase of activity over the successive postoperative days. No sustained vigorous intensity activity was recorded in either SLNB or ALND groups. There was a statistically significance difference between light activity performed preoperatively and on postoperative day (POD) 1–14 (*p* < 0.001) for SLNB and on POD 1–9 (*p* < 0.05) for ALND. There was no statistically significance difference between the light activity performed preoperatively and on POD 10–14 for ALND. There was a statistically significant difference between moderate activity performed preoperatively and on POD 1–10 (*p* < 0.05) for SLNB and POD 1–14 (*p* < 0.05) for ALND. There was no statistically significance difference between moderate activity performed preoperatively and POD 11–14 for SLNB (Fig. 4a and 4b).

### Correlation of PA with DASH and EQ-5D-5L Questionnaire

A moderate negative correlation was identified between PA level of the surgically treated side and the DASH score in week 1 (*R* = −0.4, *p* = 0.01) and a weak positive correlation between PA level of the surgically treated side and the EQ-5D-5L score (*R* = 0.33, *p* = 0.37) in week 1.

### Analgesia Requirement and Correlation of PA (Surgically Treated Side) with Pain Score

All patients in the study were prescribed regular analgesia and were compliant with their prescribed analgesia. Patients who underwent ALND were found to have a higher analgesia requirement (need as required analgesia at least once on top of the regular analgesia) compared with those who underwent SLNB (23.6% versus 6.6%). There was a weak negative correlation between PA level and pain scores (*R* = −0.283, *p* = 0.06) (Supplementary Fig. 4a) and a weak negative correlation between PA level and pain on movement score (*R* = −0.357, *p* = 0.015) in week 1 (Supplementary Fig. 4b). Additionally, there was a very weak correlation between PA level and pain score (*R* = −0.208, *p* = 0.341) (Supplementary Fig. 4a) and no correlation between PA level and pain on movement score (*R* = −0.031, *p* = 0.89) in week 2.

### Complications

Two patients developed seroma, one had an infected hematoma, and another patient in the SLNB group experienced cording, whereas two patients developed seroma and one patient had an abscess in the ALND group.

## Discussion

This study gives new insights into the objective measurement of the impact of ALND and SLNB as well as the importance of axillary de-escalation to reduce physical morbidity. ALND is associated with objectively greater physical morbidity compared with SLNB when comparing the activity level on the surgical side, particularly in the second postoperative week. This notwithstanding, while comparatively the physical convalescence following SLNB is favorable compared with ALND, we observed that SLNB impacts PA levels even up to 2 weeks after surgery.

Our findings are consistent with the ALMANAC trial,^14^ which found that after a 1-year follow-up, ALND had higher arm/shoulder morbidity and lower QoL than the SLNB group. Our meta-analysis^3^ showed that there was a 13.7% difference in the prevalence of lymphedema between ALND and SLNB. The prevalence of lymphedema after ALND at > 24 months can be as high as 30.9%. Additionally, the rate of pain, reduced ROM, and reduced strength were found to be higher after ALND than SLNB.^3^ Axillary de-escalation is driven by a desire to prevent UL damage and an increasing understanding of the oncological safety of axillary conservation.^21^

However, SLNB alone has significant complications, as demonstrated in the current study, in which a decrease in PA level over 2 weeks postoperatively that did not return to baseline level was observed. Even though our findings are not unforeseen, we would have anticipated that patients who underwent SLNB would reach their baseline level 2 weeks following surgery.^14^ A recent meta-analysis found that the lymphedema rate, pain, reduced ROM, and reduced strength can be up to 8%, 22%, 20%, and 15%, respectively, after SLNB surgery.^3^ Similarly, in the B-32 trial, the incidence of lymphedema was 7.5% and the prevalence of sensory and other mobility impairments ranged from 5% to 8%.^22^ These findings suggest the need to explore non-invasive alternatives to SLNB, such as microbubbles^23^ or super resolution ultrasound,^24^ as well as perform an adjunct procedure to reduce the rate of UL complications, such as axillary reverse mapping (ARM),^25^ and/or explore cases where it may be safe to abandon SLNB altogether.^26^ The lymphedema rate was observed to be as low as 3.3% on the basis of the pooled estimates of four studies in the first 12 months after surgery when ARM was performed.^3^ The much anticipated results from the SOUND trial may prove the futility of SLNB in patients with low-risk breast cancer, and therefore may eliminate unnecessary surgeries.^27^

Interestingly, there was no significant difference in overall activity levels between SLNB and ALND. It is an intriguing observation because it demonstrates that a random activity monitor or smart phone may not be sufficient to detect changes in the arm’s activity level (i.e., we have a sensitive outcome measure).

The current study suggests that patients spent the majority of their 24-h period in a sedentary state. This finding is consistent with a study that found that breast cancer survivors spent 66.4% of their waking time sedentary, 31.1% in light activity, and 2.6% in moderate to vigorous physical activity (MVPA).^28^ In the current study, there was also a significant decline in light- and moderate-intensity activities from preoperative to postoperative period for both SLNB and ALND. Many cancer survivors have PA levels that are below the recommended MVPA, which is associated with more complications and a reduced QoL.^29–32^ A study showed that WAMs could increase their MVPA time and this behavior is sustainable, at least in the short term.^33^ Bruce et al.^34^ demonstrated that structured exercise programs were clinically effective at reducing UL morbidity in individuals at risk of developing complications 1 year following breast cancer treatment. In addition to increasing overall activity levels, the exercise program investigated in the PROSPER study included shoulder strength and mobility exercises. As assessed by the DASH questionnaire, the improvement in UL function occurred without a change in overall PA levels.^34^ WAMs can be combined with exercise regimens so that clinicians can monitor PA levels and provide real-time feedback. Wrist-worn activity monitors can send behavioral cues or motivational messages to encourage patients to do the recommended exercises.^35^ If implemented correctly, WAMs have the potential to enhance patient satisfaction, cost efficiency, and functional outcomes.

Although there was a significant difference in PA level in week 2, the impact of ALND may have been underestimated due to the heterogeneity of the patients in our cohort. Reconstruction procedures made up a higher percentage in the SLNB cohort compared with the ALND cohort (40% versus 28.6%), which would be associated with greater postoperative morbidity and possibly lower PA levels. There was a significant difference in PA level in week 2 after eliminating heterogeneity through subgroup analysis of Mx-SLNB versus Mx-ALND, which showed the true impact of ALND. When patients without drains were excluded from this subgroup, similar findings were observed. There was no discernible difference in activity levels between DIEP-SLNB and DIEP-ALND. This is likely due to the significant morbidity from the abdominal component of the DIEP surgery, masking any more subtle changes from the axillary procedure. Along with axillary procedures, DIEP may also have a significant impact on arm function, where 14.1% of patients who underwent DIEP experienced shoulder morbidity.^36^

Patients in the ALND group had higher analgesic requirements than those in the SLNB group. This finding was expected, since those undergoing ALND were found to have greater pain post-surgery compared with those undergoing SLNB.^3^ There was also a weak correlation between the PA of the surgically treated side and the pain score on movement in week 1 and no correlation in week 2. This finding suggests that in the first postoperative week, pain impacts PA level. However, pain does not impact PA levels in week 2. This might indicate that the greater reduction in PA level following ALND compared with SLNB was influenced by iatrogenic morbidity. It is important to note that the type of mastectomy or reconstruction surgery could also influence pain. In this study, patients who had DIEP required more analgesia than patients who had Mx (44% versus 20%; Supplementary Data 1). This suggests that patients who had DIEP may experience more pain, which may affect their PA levels. Nevertheless, we tried to account for the impact of Mx and DIEP on the activity level by doing subgroup analyses.

Long-term data are important to ascertain how long it takes for patients to recover from different types of breast cancer and reconstructive breast surgeries. It is essential to develop a methodology that maximizes compliance while acquiring sufficient data. There is little incentive for patients to wear devices in this study due to lack of direct feedback, which is necessary to prevent bias.^3^ Intermittent review of patient feedback has led to minor amendments to improve wearability. Our compliance rate is approximately 81% (Supplementary Data 2), which is above average for this type of study.^1,2^ We are currently extending the scope of the study to acquire data at additional long-term intervals.

This study has limitations, including selection bias due to exclusion of patients with a language barrier and possible better engagement of younger patients due to familiarity with technology. To minimize this, attempts were made to communicate through an interpreter, and demonstrations of the simplicity of wearing WAMs to encourage patients were shown. In addition, the COVID-19 pandemic impacted patient recruitment, resulting in a 5-month pause in the study. Generalizability of findings may have been affected due to patients recruited from a single center and small number of patients recruited into the study, however, the demographics of this cohort are similar to those described in national audits.^37,38^ Although blinding was not possible in this study, patients were invited in a systematic consecutive manner to minimize study design bias in the data collection. While subgroup analyses were conducted to reduce confounding from concurrent procedures, the small number of patients per subgroup may result in type II error. Attrition bias may have occurred due to more patients with arm problems withdrawing/being lost to follow-up, which would underplay morbidity. This was minimized by sensitively encouraging patients to continue participation and personalized patient contact via phone. Handedness, adjuvant therapy, presence of drains, and pain could all influence PA levels. By collecting preoperative data and comparing postoperative PA levels to baseline, the effect of handedness and adjuvant therapy could be minimized. Subgroup analysis was also performed to exclude patients without drains, and trends in PA levels were commensurate with the primary analysis. Future work includes recruiting a larger number of patients and extending follow-up to understand their PA levels in the long term.

## Conclusions

WAMs could be used as a new objective tool to measure UL morbidity after breast surgeries. Compared with SLNB, ALND increases morbidity, which manifests as a decrease in arm movement as measured by WAMs. The findings also demonstrate the longitudinal impact of SLNB, which impacts PA levels, even up to 2 weeks after surgery. This is an important finding because it suggested protracted morbidity even in de-escalated surgical procedures. This information is crucial for patient counseling and for helping in the design of new trials. Non-invasive alternatives and adjunctive procedures to lower the rate of UL complications, or safely forgoing nodal mapping entirely, should be explored. Additionally, monitoring recovery objectively after axillary surgeries could be used to improve outcomes by identifying vulnerable patients who would benefit from early exercise intervention, encouraging PA, and keeping track of individualized PA that could be added to the feedback rehabilitation care plan.

### Supplementary Information

Below is the link to the electronic supplementary material.

Supplementary file1 (DOCX 536 KB)
