# Supplementary material for: The Use of Wearable Activity Monitors to Measure Upper Limb Physical Activity After Axillary Lymph Node Dissection and Sentinel Lymph Node Biopsy
Source: Ann Surg Oncol. 2023 Jul 28;30(12):7036–45. doi: 10.1245/s10434-023-13966-7 (PMC10562272; doi:10.1245/s10434-023-13966-7)
Supplement: Supplementary file 1 — Supplementary file1 (DOCX 536 KB) [file 10434_2023_13966_MOESM1_ESM.docx]

**Supplementary Online Content**

**Supplementary Table 1.** Inclusion and exclusion criteria

**Supplementary Method 2.** Study protocol

**Supplementary Table 3.** Surgically treated side only - Comparison of simple mastectomy (Mx) + sentinel lymph node biopsy (SLNB) vs Mx + axillary lymph node dissection (ALND) at week 1 and week 2 [excluding patients without drains]

**Supplementary Figure 4a**. Correlation of physical activity (PA) with pain score in week 1 and week 2

**Supplementary Figure 4b**. Correlation of physical activity (PA) with pain score on movement/specific activities in week 1 and week 2

**Supplementary Data 1.** Analgesia requirements for patients who had DIEP and Mx (+ axillary surgeries)

**Supplementary Data 2.** Compliance to WAMs

**Supplementary Table 1.** Inclusion and exclusion criteria

| **Inclusion** | **Exclusion** |
| --- | --- |
| Breast cancer patients undergoing any form of breast/and or axillary surgery* | Patients with a movement disorder (e.g. Parkinson’s Disease) |
| All age groups | Patient using mobility device or aids |
|  | Patient with inadequate comprehension of paperwork unable to provide appropriate informed consent** |

* To include mastectomy, implant and deep inferior epigastric perforator construction +/- axillary lymph node dissection or sentinel lymph node biopsy

** After attempted translation/explanation via staff or family members

**Supplementary Method 2.** Study protocol


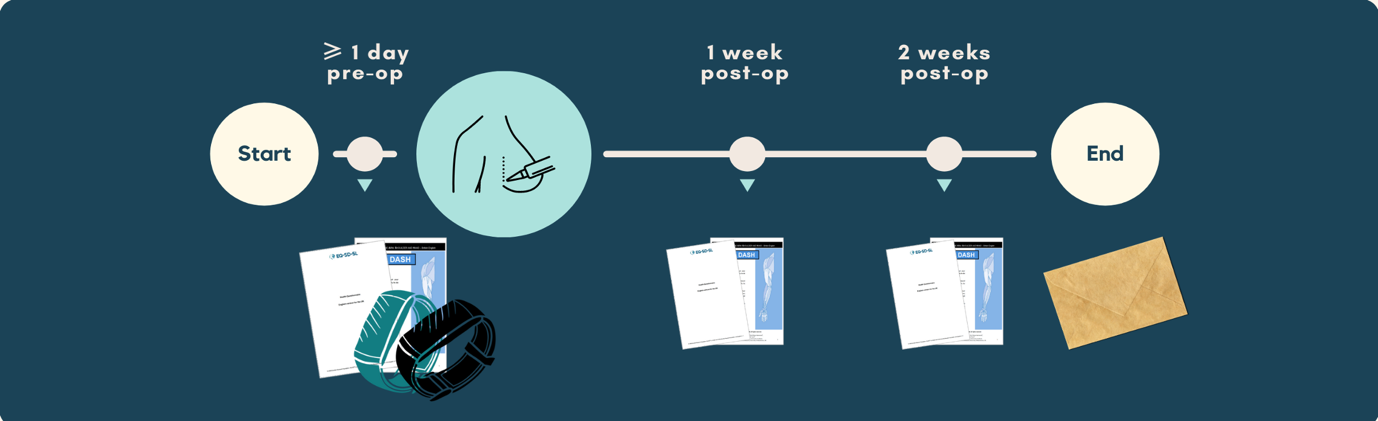


Patients were informed about the research study by the direct healthcare team in outpatient clinics. Eligible patients were approached by the research team. Following expression of interest and verbal consent, the patient was given a study information sheet and the protocol was explained. Written informed consent was gained from all study participants. There was no financial incentive offered to participate.

Patients wore WAMs on both wrists daily for an average 3 days^1-3^ prior to surgery and for up to two weeks after surgery. They were instructed to wear the WAMs 24 hours a day, although could remove them for sleeping and showering. The patients were informed that the sensors measured arm activities, but no exercise goals were provided. However, it is routine procedure in our unit to provide all patients with standard postoperative arm mobility advice and a Breast Cancer Care pamphlet on postoperative exercises [accessible at [www.breastcancernow.org](http://www.breastcancernow.org)]. Patients completed the disability of arm, shoulder and hands (DASH)^4^ and EuroQol-5D-5L (EQ-5D-5L)^5^ questionnaires before and after surgery (week 1 and 2).

The patients were instructed to begin arm mobilization the day following surgery, if feasible, and to continue doing so gradually until they had regained their pre-surgical range of motion. If they experienced seroma, wound infection/healing issues, or discomfort that intensified during these exercises or persisted after they completed them, they were instructed to discontinue the activity immediately or consult a doctor.

The DASH questionnaire consists of 30 items that assess the function of the upper limbs, with scores ranging from 0 to 100. The lower the DASH score the less the disability.^4^ The EQ-5D-5L questionnaire is a validated instrument with a descriptive component and a visual analogue scale (VAS) that may be used to measure health-related QoL in a broad variety of health conditions.^5^

Upon termination of study observations, questionnaires and sensors were returned to researchers in follow-up clinic sessions or via post in prepaid envelopes. The wrist-worn sensors (AX3; Axivity, Newcastle upon Tyne, UK) are triaxial accelerometers that are commercially available and permit manual calibration, full data download, and analysis using a software. The Open Movement Graphical User Interface OMGUI (version 1.0.0.37) was utilized for calibration and computations of activity levels utilizing signal vector magnitude (SVM), a well-established approach for merging triaxial accelerometer data to create a quantifiable activity level. Pain scores were inferred from DASH questionnaires. Patients were interviewed after (or during) the period of data collection so that they could report any technical challenges encountered or if the WAM had been removed for any amount of time. Any WAMs that were lost or damaged were replaced (n =7).

**Supplementary Table 3.** Surgically treated side only - Comparison of simple mastectomy (Mx) + sentinel lymph node biopsy (SLNB) vs Mx + axillary lymph node dissection (ALND) at week 1 and week 2 [excluding patients without drains]

| **Week** | **Mx-SLNB (Median %)** | **Mx-ALND (Median %)** | **p value** |
| --- | --- | --- | --- |
| 1 | 52.8 | 55.0 | 0.321 |
| 2 | 81.6 | 69.1 | 0.023 |

**Supplementary Figure 4a**. Correlation of physical activity (PA) with pain score in week 1 and week 2

**
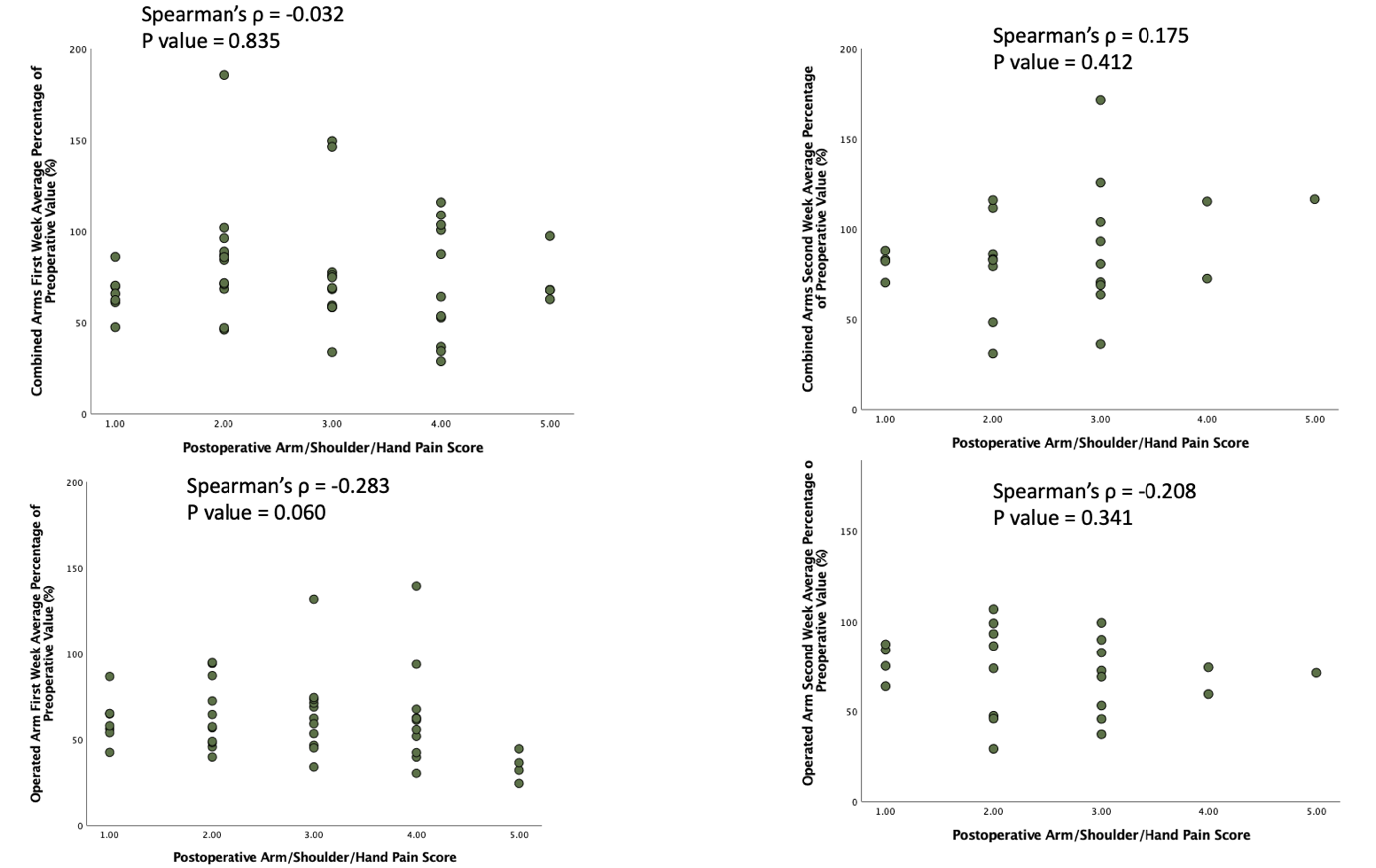
**

**Supplementary Figure 4b**. Correlation of physical activity (PA) with pain score on movement/specific activities in week 1 and week 2

**
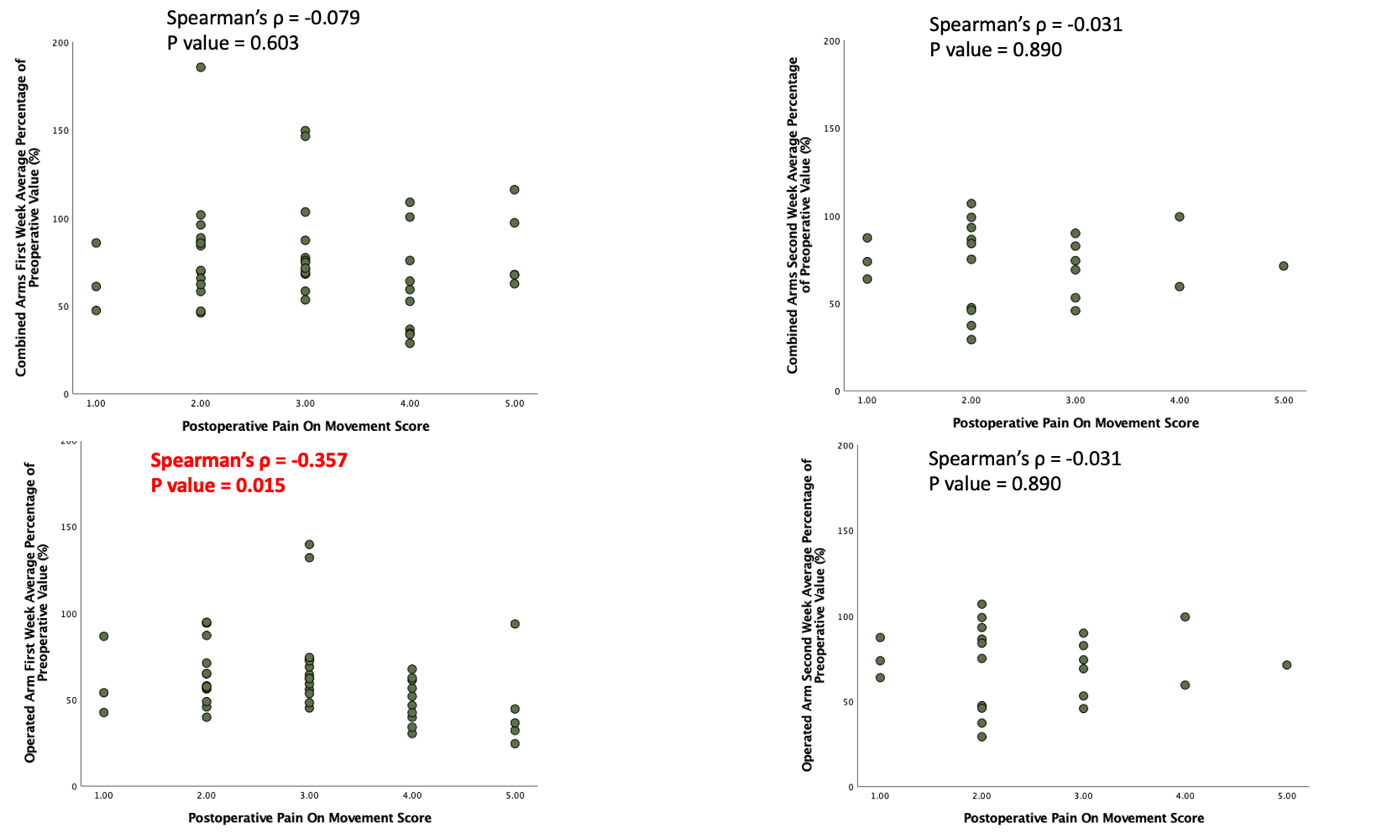
**

**Supplementary Data 1.** Analgesia requirements for patients who had DIEP and Mx (+ axillary surgeries)

DIEP patients were found to have a higher analgesia requirement (need as required analgesia at least once on top of the regular analgesia) compared to Mx patients (44% vs 20%).

**Supplementary Data 2.** Compliance to WAMs

From all the patients in the study, 784 (56 patients x 14 days) data points in the postoperative period could have been captured. Out of 784 data points, 150 data points were not captured. Therefore, the overall adherence to the WAM application protocol was 80.7%.

**References**

1. Dillon CB, Fitzgerald AP, Kearney PM, et al. Number of Days Required to Estimate Habitual Activity Using Wrist-Worn GENEActiv Accelerometer: A Cross-Sectional Study. *PLoS One.* 2016;11(5):e0109913.

2. Hart TL, Swartz AM, Cashin SE, Strath SJ. How many days of monitoring predict physical activity and sedentary behaviour in older adults? *Int J Behav Nutr Phys Act.* 2011;8:62.

3. Ricardo LIC, Wendt A, Galliano LM, et al. Number of days required to estimate physical activity constructs objectively measured in different age groups: Findings from three Brazilian (Pelotas) population-based birth cohorts. *PLoS One.* 2020;15(1):e0216017.

4. Hudak PL, Amadio PC, Bombardier C. Development of an upper extremity outcome measure: the DASH (disabilities of the arm, shoulder and hand) [corrected]. The Upper Extremity Collaborative Group (UECG). *Am J Ind Med.* 1996;29(6):602-608.

5. Janssen MF, Pickard AS, Golicki D, et al. Measurement properties of the EQ-5D-5L compared to the EQ-5D-3L across eight patient groups: a multi-country study. *Qual Life Res.* 2013;22(7):1717-1727.
